# Personalised informed choice on evidence and controversy on mammography screening: study protocol for a randomized controlled trial

**DOI:** 10.1186/s12885-017-3428-9

**Published:** 2017-06-19

**Authors:** Anna Roberto, Cinzia Colombo, Giulia Candiani, Livia Giordano, Paola Mantellini, Eugenio Paci, Roberto Satolli, Mario Valenza, Paola Mosconi

**Affiliations:** 1 IRCCS-Istituto di Ricerche Farmacologiche Mario Negri, Via G. La Masa 19, 20157 Milan, Italy; 2 Zadig - Science and Health Communication, Milan, Italy; 3 Screening Unit, Cancer Prevention and Research Institute – ISPO, Florence, Italy; 4 U.O. Centro Gestionale Screening, Azienda Sanitaria Provinciale di Palermo, Palermo, Italy; 5 LILT, Lega Italiana per la lotta contro i tumori, Sezione di Firenze, Florence, Italy; 6 GISMA, Gruppo Italiano Screening Mammografico, Florence, Italy

**Keywords:** Informed choice, Mammography screening, Decision aid, Decision-making

## Abstract

**Background:**

In Italy women aged 50–69 are invited for a population-based breast cancer (BC) screening. Physicians, policy makers and patients associations agree on the need to inform women about the benefits and harms in order to permit an informed decision. Decision aids (DA) are an effective way to support people in their decisions about health. This trial aims to assess women’s informed choices, according to their health literacy and values, on participating or not in BC screening for the first time. Benefits, harms and controversies are presented.

**Methods/design:**

The impact of the DA will be evaluated in a randomized controlled trial with a two-week follow-up. Women will be randomized via web to DA or a standard brochure. We will invite 8160 women, to obtain a final sample of 816 women. The primary outcome will be informed choice, measured on the basis of knowledge, attitudes and intentions on BC screening. Secondary outcomes are participation rate, satisfaction on information and decisional conflict.

**Discussion:**

The web DA will be open-source and implemented on BC screenings and its efficacy for increasing informed choice will be tested. This model could be applied to other healthcare settings, cancer screenings, and public health programs.

**Trial registration:**

The protocol for this trial was registered with the Clinicaltrials.gov registry on March 16, 2017: NCT03097653.

## Background

In the 1990s, several European countries implemented screening services as public health programs. In 2003, the European Council Recommendation promoted the implementation of screening programs in Europe, for breast, bowel and cervical cancer. This decision was confirmed, after assessment of the available evidence, in the recently released European code against cancer [1].

In Italy, as in other European countries, mammography screening is offered by the National Health Service (NHS), free of charge. About 80% of the target women are invited, with wide variability among northern and southern regions [2]. The policy is to invite women aged 50–69 - in some regions extended to 45–69 or 50–74 - by a personal letter, with a pre-specified appointment every two years.

Fostering informed decision-making on breast cancer screening by providing balanced information on its benefits and harms is considered a responsibility of the public health system [3].

Although the debate on mammography screening is lively [4–7], most stakeholders - physicians, policy-makers, lay people or patients’ associations - agree on the need to inform women properly, and consider this an ethical obligation [8]. The current debate on mammography screening adds a new challenge: how disagreement among scientists, such as uncertainties about the estimates especially of potential harms from the screening, should be managed, and how public health institutions and scientists should cope with communication when there are data interpretation divergences and conflicts.

Some studies have shown that women did not properly understood the value of mammography screening, confounding early diagnosis with prevention [4, 9]. Other studies have found that many leaflets and websites fail to provide all the right information to women, highlighting more the benefits than the risks, such as overdetection and overtreatment [10–12]. In 2012, the NHS-UK (United Kingdom) set up an Independent Panel to review the evidence, and concluded for the continuation of screening program, but asked for changes in the communication and information about benefits and harms, especially overdetection of breast cancer [13].

The screening promoters therefore have the duty to provide all the information relevant for an informed choice, including that on the uncertainty of the estimates and the scientific controversy, to provide to women the best benefit-risk estimate, based on professional opinions.

In cancer screening, the communication of quantitative information is particularly complex. However, transparency about benefits and harms is a key principle for good quality information – although this should hold true for any kind of information, in all human interactions [14].

Qualitative studies have discussed how to balance the evidence-based information to enable women to participate in healthcare decisions [15]. Different models of information have been suggested, and formally tested in randomized controlled trials (RCTs) [16, 17]. Decision aids (DA) are an effective way to help women to decide about mammography screening. They improve people’s knowledge of risks and benefits, reduce decisional conflict related to feeling uninformed and unclear about personal values, and encourage women to take a more active role in decision-making without anxiety [13, 14].

To give women the chance to decide according to their preferences, values, and attitude, information must be organized and delivered through a multilevel, interactive model that takes into consideration the users’ needs and willingness. This model should leave women free to seek the depth and breadth of information necessary to make a weighted decision, in a personalized way that should also respect their “right not to know”.

A personalized informative model can influence not only the participation in breast cancer screening, but also empower personal choice: increasing women’s awareness, maintaining realistic expectations, and increasing women’s satisfaction about the decision process.

This RCT is the experimental phase of a project regarding the decision process on breast cancer screening participation. The project was informed by a review of the literature on DA and the organization of focus groups to collect women’s information needs (phase 1). A web interactive DA was developed, where benefits, harms and controversy on mammography screening are fully presented (phase 2). The present study aims to assess the effect of this interactive web DA on informed choice – measured on the basis of knowledge, attitudes and intentions concerning breast cancer screening – comparing the DA with standard information provided on the web.

Secondary aims are the participation rate in breast cancer screening, satisfaction with the information, the time spent on the DA, and the decisional conflict process.

## Methods

This is a multicenter RCT. The hypothesis is of superiority for the primary endpoint regarding the efficacy of the DA in increasing women’s informed choices. If the null hypothesis is rejected, the secondary endpoint regarding the screening participation rate will be analysed with a non-inferiority hypothesis. The project is funded by AIRC, the Italian Association for Cancer Research - IG2015–17274.

### Study setting

The study has been implemented within the National Health Service screening program in Turin [18], Florence [19] and Palermo [20], respectively in the north, center and south of Italy. Organized mammographic screening in the Florence Health District in the Tuscany Region has been ongoing since the late 1980s. Every two years it invites 50–69 year-old women and the 70–74 year women who had participated in the previous round. Recently the Tuscany Region Government decided to gradually implement the organized screening for 45–49 year-old women, inviting them annually until age 50.

In Turin, organized mammographic screening was introduced in 1992. Every two years it invites 120,000 50–69 year-old residents. Currently women aged 45–49 receive an informative letter, giving them the opportunity to spontaneously join the program, and have an annually mammogram. Women aged 70–74 years can also agree to continue to participate in the biennially program. In Palermo organized mammographic screening started in 2004, inviting 86,000 50–69 year-old women every two years.

The participation rate among is about 50% for Florence, 75% for Turin and 40% for Palermo.

The programs have a consolidated monitoring and quality assurance system, that collects and analyses data on a yearly basis.

### Participants’ eligibility and randomization

Newly invited women to the three screening programs (Florence, Palermo and Turin) will be invited to participate in the trial. Since of all three screening programs are quite old, the number of newly invited women at every round is limited, accounting for, at most, 25% of the entire 50–69 year-old subjects. The largest group is mainly the 50–52 year-olds. In this trial, the majority of women enrolled will probably belong to that particular age group and to 45–49 group for the two centers that implemented screening from 45 to 74. Women of this age in each screening center, will receive an invitation letter to the trial with a personal code number for registering on the platform. All code numbers will be extracted and transferred from the screening centers to the platform. The random allocation will be on a 1:1 basis, provided by a computer-generated allocation sequence. No stratification will be done. The nature of the intervention and allocation ratio precludes masking of the participants and trial staff.

### Intervention and control arms

**Fig. 1:**
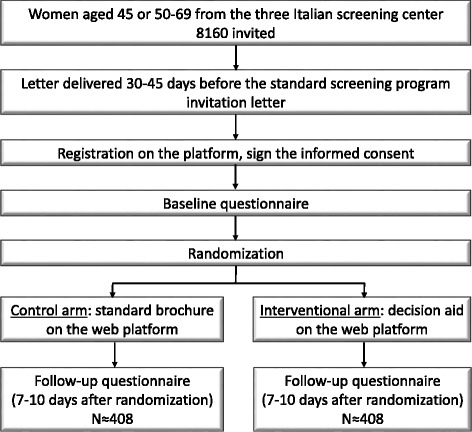
Study flow diagram
- Control group: Web platform with a standard brochure.

The women will be randomized to (Fig 1):

- Intervention group: Web platform with multilevel information and an aid for the decision to be taken.

#### Intervention

The content in the web platform is structured in 16–20 screens; each screen contains the answer to a question (i.e. What is breast cancer? What is mammography screening? What are its benefits and harms? What results can be expected from mammography screening?). The language is plain and the contents are defined on the basis of the literature. Papers and systematic reviews [21–25] available in the literature, reports of institutional organizations and guidelines, other screening materials (leaflets, website, brochure) are carefully assessed in order to collect all the information needed for a balanced and honest tool. The information also covers controversial topics such as overdetection, overtreatment and the disagreement among scientists about quantification of harms and benefits.

The navigation is personalised, with a “nudging” approach that induces women to become informed on the main matters. However, women can stop reading whenever they feel ready to decide. When woman feels she knows enough and is to decide the platform provides a DA module listing issues and concerns that can affect their decision. Women are asked to state, the importance of each items, and its impact on their decision. The quantitative data presented in the DA come from the UK Panel (RCTs) and from the EUROSCREEN benefit-harms ratio (breast cancer mortality, overdetection and false-positive), based on an observational study assessing screening program outcomes in Europe. The controversy is presented based on the quantitative estimates of the 2013 Cochrane Review [4].

#### Control

The standard brochure combines the best information from the three participating centers’ brochures. Numbers of lives saved thanks to screening, false-positive cases, and over-diagnosed tumors are reported. The brochure gives no information on the controversy about mammography screening.

### Consent and data collection

Eligible women will be invited to participate by their screening center through an ad-hoc letter providing information regarding the trial and how to access the web platform. After 30–45 days, the standard screening invitation, reporting the appointment for mammography, will be sent to women with a reminder letter to solicit participation in the trial. Women agreeing to participate will enter the web platform, register themselves, sign informed consent and complete a baseline questionnaire covering the topics reported in Table 1. After that, they will be randomized to the intervention or the control group. Women who do not complete the questionnaire will be reminded by e-mail one week after they registered and signed informed consent.

**Table 1:** Schedule of enrolment, variables and measures

Timepoint		Trial period	
	Enrollment	Allocation	Post-allocation
	Baseline	0	Post (7–10 days after randomization)
Registration and signed informed consent	X		
Allocation		X	
Demographics	X		
Use of internet	X		
Experience with other screenings	X		
Previous mammography	X		
Previous participation in screening programmes	X		
Family history of breast cancer	X		
Perceived risk of breast cancer	X		
Knowledge of breast cancer screening	X		X
Attitude to breast cancer screening	X		X
Intention regarding breast cancer screening	X		X
Satisfaction and acceptability of the information received			X
Decisional conflict			X

Seven to ten days after randomization, a link to a follow-up questionnaire will be sent to participants (Table 1). Finally, a reminder link and thankyou will be emailed to all women who initially agreed to participate.

### Study endpoints

In order to draft the questionnaires related to the primary and secondary endpoints, a systematic review of the literature was done, searching for RCTs on DA dealing with mammography screening. Two independent reviewers examined the literature, and nine trials were included. A comparative analysis of the tools used in these trials was presented and discussed with partners and the scientific committee, identifying areas and items for consideration.

The questionnaire items were translated into Italian through a multistep process employing standardized methodology. Briefly, one professional translator and two members of the coordinating center (AR, CC) produced three independent Italian translations. After discussion, a shared Italian version was produced. This version was then evaluated by partners (epidemiologists with experience in breast cancer screening, experts in communication, researchers) in terms of the use of simple, correct language.

The preliminary Italian version of the questionnaires was tested on a small group of women in order to evaluate its clarity, understandability and length. At the end of this, a final version was established.

#### Primary endpoints

Informed choice will be measured according to the three-dimensional framework of Marteau et al. [26], which covers knowledge, attitude and intention. The statistical hypothesis for the primary endpoint is superiority. The trial is designed to detect a 10% difference between the control and the intervention group, according to the literature [24, 25].

Knowledge will be measured using a questionnaire developed on the basis of literature [24, 25] structured in 13 questions with multiple-choice answers, with two to three options. Ten questions are qualitative and three numerical. Each correct answer will receive one point. The maximum total score is 13 out of 13. The score and threshold to reach “adequate knowledge” were decided beforehand, following the approach described in the literature [24, 25]. A score of 8 out of 13 (about 60%) or higher will be considered “adequate knowledge”.

Attitude will be measured on a scale used in the literature [24, 25], consisting of six items with five response options from 1 to 5, with a total score from 6 to 30. For informed choice, we set the threshold for a positive attitude at 24, and so a score below <24 means a negative attitude.

Intention to be screened will be measured using one item with five responses: Definitely will, Likely to, Unsure, Not likely to, Definitely will not. For informed choice, we classified “definitely will” and “likely to” as positive intentions. This item will be collected both at the end of the information session on the web, and in the follow-up questionnaire. If there is no consistency between the two answers, the answer to the follow-up questionnaire will be considered conclusive.

Informed choice will be assessed as a dichotomous outcome. A woman with adequate knowledge and consistent attitudes and intentions (positive or negative) will be considered as expressing informed choice.

#### Secondary endpoints

The participation rate in the breast cancer screening program will be assessed as the percentages of women who participate in the trial, in the intervention and the control groups. The statistical hypothesis for the secondary endpoint is non-inferiority.

Satisfaction with the information given (intervention and control group) will be measured using eight items regarding length, quantity, clarity, balance, helpfulness of the information in making a decision, and willingness to recommend it to other women, with a three-point scale.

The time spent on the pages in the web DA and the web brochure will be assessed with Pickwick software. Only for the web DA, the number of pages visited, frequency of access and level of detail reached will be calculated with the same software.

Decisional conflict will be assessed using the validated and widely used Decisional Conflict Scale-SURE version [27]. This four-item scale will assess the woman’s knowledge of the options available, clarity about the benefits and risks most important for them, adequate level of support, and conviction about the best choice. Since no Italian translation of this scale is available, the scale will be translated for this trial.

The perceived risk of breast cancer will be assessed using one item with five verbal response categories ranging from “much lower” to “much higher” than the average.

### Sample size

The primary analysis will compare the proportion of women who make an informed choice, using the chi-squared test in the two study groups. Based on previous studies [22, 25] we judge an absolute difference of 10% as the minimum important difference for the sample size calculation. Assuming that one of the group proportion is 50%, in order to achieve 80% power to detect a group difference with a two-sided significance level of 5%, we require 816 women at follow-up. Allowing for an estimated response rate of 15% and early drop-out of one-third of initial participants, we will invite 8160 women to take part.

If the null hypothesis related to the primary endpoint is rejected, the first of the secondary endpoints will be analysed with a non-inferiority hypothesis. The power of the analysis for this non-inferiority test - with one-sided tail - will be considered depending on the participation rate at breast cancer screening.

### Data analysis

We will conduct a descriptive analysis for the trial participants. Possible baseline differences between trial arms will be statistically tested.

For the primary endpoint, statistical analysis will be done on an intention-to-treat basis: all the women randomised, compliant to follow-up will be included in the analysis in the group assigned at randomization. The impact of the web DA on the primary endpoint will be analysed using the chi-square test.

For secondary endpoints, we will use the chi-square test to analyse binary endpoints and a two-sided t-test for continuous endpoints, with a significance level of 5%. We will use SAS statistical software, version 9.2.

### Ethical approval, dissemination and trial registration

The study will be conducted in accordance with the principles of the Declaration of Helsinki. Ethical approval was obtained from the ethics committee of the coordinating center, and from the ethics committee of each participating center. Participants will give online informed consent after reading complete, clear information regarding the nature and purpose of the trial. Any modifications or amendments that affect the conduct of the trial will be documented, resubmitted for approval to the ethics committees, and reported in further publications. To ensure data privacy, confidentiality will be assured by coding each women enrolled, through assignment of a unique identification number.

The trial is registered with the ClinicalTrials.gov registry (NCT03097653) on March 16, 2017.

Results will be published in a public registry (Clinicaltrials.gov), in peer-reviewed scientific journals, and disseminated to lay people. According to the recommendations of the International Committee of Medical Journal Editors only persons directly involved in the trial will be designated as authors.

## Discussion

This project will develop a new model for providing high-quality health information to women invited to participate in breast cancer screening. The model employs an interactive process in which the woman can choose the kind and depth of information she wants over time, and covers topics highly recommended by women, researchers and public entities for to communicating the benefits and harms of screening. These will include over-diagnosis and controversies regarding breast cancer screening, scantily covered in communications aimed at women invited to participate in screening.

An open-source software for a web platform draws on innovative information and communication technologies, incorporating all the features and functions in the model. The RCT will test the efficacy of this web DA in increasing informed choice on breast cancer screening, providing a tool which, if valuable, could be used in the future by women in other Italian screening programs.

The model, the software and the web platform will be free of charge and publicly usable (under a creative common licence).

The transferability of the model and platform to other settings, other countries and other cancer screenings (for example, colon cancer screening) could be assessed as a further step.

### Pitfalls and caveats

The proposed Web platform with its multilevel information may be too innovative for the target women, and this could influence participation in the trial. Recent data, however, show that about 60% [28] of the Italian population has regular access to the web, and this continuously increases. This figure helped us decide to develop a web platform to inform women. In this trial, women aged 45–52 years will be enrolled, and may be less representative of the older segments of the population. Nevertheless, with the large number of women involved in the screening, the trial should in any case recruit a significant number of cases.

There is also the possibility that the trial falsifies the hypothesis and does not show any superiority of the Web DA over the Web platform brochure. However, the trial will collect a rich set of data that will be useful to improve the new model.

## Abbreviations

AIRC: The Italian Association for Cancer Research
BC: Breast Cancer
DA: Decision Aid
NHS: National Health Services
RCTs: Randomized controlled trials
UK: United Kingdom
